# Integrating Sleep, Eating, Bowel Habits, and Physical Activity in Children and Adolescents: A Conceptual Review of the Four Kai Framework

**DOI:** 10.3390/children13081115

**Published:** 2026-08-20

**Authors:** Jun Kohyama

**Affiliations:** Tokyo Bay Urayasu Ichikawa Medical Center, 3-4-32 Toudaizima, Urayasu 279-0001, Chiba, Japan; info@j-kohyama.jp

**Keywords:** children, adolescents, bowel habits, diet, physical activity, sleep health, chrono-nutrition, daytime functioning, self-regulation

## Abstract

**Highlights:**

**What are the main findings?**

- Recent pediatric evidence supports multiple associations among sleep, eating, physical activity, and daytime functioning, whereas evidence regarding everyday bowel habits remains comparatively limited.
- The Four Kai framework integrates sleep, eating, bowel habits, and physical activity and proposes adaptive pleasure and positive self-regulation as hypothesis-generating concepts linking these behaviors with daytime functioning.

**What are the implications of the main findings?**

- Pediatric health may benefit from considering sleep together with eating, bowel habits, and physical activity rather than addressing these daily physiological behaviors entirely in isolation.
- Longitudinal and intervention studies are needed to determine whether the Four Kai behaviors are functionally interconnected and whether adaptive pleasure and positive self-regulation contribute to healthy daytime functioning.

**Abstract:**

Background/Objectives: Recent pediatric sleep research has increasingly adopted an integrated lifestyle perspective, examining sleep together with diet, physical activity, sedentary behavior, and family environments. However, bowel habits remain comparatively underrepresented despite their potential importance for child health. This conceptual narrative review revisits the Four Kai framework, which brings together sleep, eating, bowel habits, and physical activity as four fundamental daily physiological behaviors and explores their potential relationships with adaptive pleasure, self-regulation, and daytime functioning. Methods: This conceptual narrative review incorporated a descriptive PubMed literature-mapping exercise conducted on 16 July 2026. The search covered publications from January through June of each year from 2022 to 2026 and focused on studies in which sleep was a principal research topic together with terms related to diet, bowel function, physical activity, and lifestyle. Publications clearly outside pediatric or adolescent populations were excluded. Titles and abstracts were reviewed, with full texts consulted when necessary, and eligible studies were classified thematically according to their principal research objective. This exercise was intended to provide descriptive context for the conceptual review and was not conducted as a systematic or scoping review. Results: The descriptive mapping and narrative synthesis indicate that pediatric sleep research frequently examines sleep in relation to diet, physical activity, sedentary behavior, family environments, and other lifestyle factors, whereas everyday bowel habits remain comparatively underrepresented. The reviewed literature supports multiple individual and pairwise associations among sleep, eating, physical activity, bowel function, and daytime functioning but does not establish that these four behaviors operate as a single integrated physiological system. Direct empirical evidence for a pathway linking the Four Kai through adaptive pleasure and positive self-regulation to daytime functioning is currently lacking. Conclusions: The Four Kai framework is best regarded as a hypothesis-generating conceptual framework rather than an established biological or causal model. Its potential value lies in bringing sleep, eating, bowel habits, and physical activity into a common perspective and generating testable questions about their possible functional integration. Future longitudinal and intervention studies should evaluate these behaviors together with adaptive pleasure, self-regulation, and daytime functioning to determine which proposed relationships can be empirically supported.

## 1. Introduction

Drawing on more than three decades of clinical experience in sleep medicine together with the remarkable expansion of sleep research over recent decades, the author introduced the concept of the Four Kai in 2011, which integrates sleep, eating, bowel habits, and physical activity as four fundamental physiological behaviors [1].

The Japanese word “kai” (快) refers here to a pleasant, refreshing, or relieving subjective state rather than pleasure in the purely hedonic sense. In the present framework, adaptive pleasure does not refer to pleasure per se or to the immediate hedonic enjoyment of a behavior. Rather, it refers to such a subjective state when associated with the appropriate fulfillment of a physiological need and the restoration or maintenance of homeostasis. Accordingly, hedonic pleasure arising from a behavior that does not support physiological regulation—for example, excessive consumption of highly palatable foods—is not necessarily regarded as adaptive pleasure.

Kai is retained as a culturally specific term because no single English word simultaneously captures refreshment, physiological restoration, and adaptive subjective reward. Throughout this review, adaptive pleasure is used as the closest English approximation of the concept. The Four Kai framework is not intended to encompass all forms of human pleasure. Rather, it focuses on four fundamental daily physiological behaviors—sleep, eating, bowel habits, and physical activity. Adequate sleep is fundamental to health and daytime functioning throughout development [2], while healthy physiological and behavioral patterns established during childhood may have implications across the life course [3]. Within the proposed framework, the appropriate fulfillment of the four behaviors is hypothesized to be accompanied by distinct forms of adaptive pleasure associated with the restoration or maintenance of biological homeostasis.

The present review views adaptive pleasure not merely as a subjective experience resulting from appropriate physiological behaviors but also as a possible signal associated with physiological regulation. Such experiences may contribute to the repetition of healthy behaviors through self-regulation. However, this proposed pathway should currently be regarded as a conceptual hypothesis, as direct empirical evidence supporting the complete causal mechanism remains limited.

In this review, daytime functioning refers broadly to cognitive performance, academic achievement, emotional regulation, alertness, social functioning, and health-related quality of life. Adequate sleep is associated with multiple aspects of physical, cognitive, emotional, and daytime functioning in children and adolescents [4]. Insufficient sleep during adolescence is associated with adverse daytime, behavioral, and health consequences [5], and insufficient sleep is a major clinical concern in pediatric sleep medicine [6]. Insufficient sleep syndrome has also been specifically highlighted as an important sleep-related problem during childhood [7]. Eating, bowel function, and physical activity may likewise be associated with different aspects of daytime functioning, as reviewed in the subsequent sections; however, the strength of evidence differs substantially among these domains.

Despite the growing emphasis on integrated lifestyle approaches, an important conceptual gap remains. Existing frameworks have primarily focused on combinations of sleep, diet, physical activity, sedentary behavior, screen use, and family or environmental influences, often within the framework of 24-h movement behaviors and related lifestyle factors [8,9]. Notably, recent mapping of integrated 24-h movement research has focused primarily on physical activity, sedentary behavior, and sleep, with everyday bowel function not included as a component of these models [10]. This relative lack of attention to everyday bowel function was also reflected in the descriptive literature mapping conducted in the present review. Moreover, many studies have investigated these domains individually or in limited combinations, and it remains unknown whether sleep, eating, bowel habits, and physical activity are functionally interconnected in children and adolescents. The novelty of the Four Kai framework is that it identifies bowel habits as a potentially relevant yet underexplored component within contemporary integrated lifestyle research, while bringing these four fundamental daily physiological behaviors into a single hypothesis-generating framework and proposing adaptive pleasure and positive self-regulation as possible processes linking these behaviors with daytime functioning. In this sense, the Four Kai framework is intended to complement existing concepts such as sleep health and integrated lifestyle behaviors while generating new, testable questions for pediatric research.

Accordingly, this narrative review summarizes recent pediatric and adolescent sleep research from the perspective of the Four Kai framework and examines whether and how these four physiological behaviors might be considered within an integrated perspective on healthy daytime functioning. Rather than proposing a new evidence-based causal model, this review aims to provide a conceptual framework for integrating findings from research fields that have traditionally evolved independently and to identify priorities for future longitudinal and intervention studies. This article should therefore be viewed as a conceptual narrative review that synthesizes recent evidence and uses the Four Kai framework to generate testable questions for future research.

## 2. Recent Trends in Pediatric and Adolescent Sleep Research

### 2.1. Search Strategy and Descriptive Literature-Mapping Procedure

Research on sleep in children and adolescents has undergone remarkable expansion over the past four decades. Using 1986—the year in which the author’s first English-language paper on pediatric sleep was published [11]—as a historical reference point, the evolution of the field was first examined through a PubMed search. The PubMed database was searched using the query sleep[Title] AND (child OR adolescen*) for articles published between January and June 1986. A total of 62 publications were identified during this period. In contrast, the same search strategy performed on 16 July 2026 at 11:15 JST identified 1516 publications published between January and June 2026. This approximately 25-fold difference illustrates the substantial expansion of pediatric and adolescent sleep research over the past four decades.

To characterize recent research themes relevant to sleep and the Four Kai framework, a separate descriptive literature-mapping exercise was conducted using PubMed. This exercise was intended to provide contextual information for the present conceptual narrative review rather than to constitute a systematic review, scoping review, or bibliometric analysis. Because of this descriptive purpose, the search was limited to PubMed. The search was performed on 16 July 2026 at 11:15 JST and covered publications from January through June of each year between 2022 and 2026. The five-year period beginning in 2022 was selected as a pragmatic contemporary window to characterize recent research themes relevant to the Four Kai framework, rather than to provide a historical or exhaustive review of the pediatric sleep literature. The starting year of 2022 was not selected because of a specific change in the field but rather to define a sufficiently broad contemporary period for describing the current research landscape. Because publications for 2026 were available only for the first half of the year at the time of the search, the January–June period was applied consistently to all five years to allow comparison across equivalent six-month observation periods.

The exact PubMed search string was: sleep[Title] AND (child OR adolescen*) AND (breakfast OR dinner OR meal OR eating OR diet OR nutrition OR bowel OR constipation OR defecation OR stool OR gut OR microbiome OR microbiota OR lifestyle OR “health behavior” OR “physical activity” OR exercise).

For each year from 2022 through 2026, the PubMed publication-date filter was applied separately to restrict the search to publications dated from 1 January through 30 June. No separate MeSH-based search was performed, and no language restriction was applied. The search was intentionally restricted to publications containing “sleep” in the title in order to identify studies in which sleep represented a principal research focus rather than a secondary or peripheral variable. This approach increased search specificity but reduced sensitivity and may have excluded relevant studies in which sleep was mentioned only in the abstract. The search initially identified a total of 1579 publications: 260 in 2022, 259 in 2023, 333 in 2024, 365 in 2025, and 362 in 2026. Studies were eligible if they primarily focused on children or adolescents and addressed sleep-related outcomes. Studies were excluded if they clearly did not primarily involve children or adolescents. No additional language-based exclusion criterion was applied. After these exclusions, 1196 publications remained for thematic classification: 185 in 2022, 217 in 2023, 247 in 2024, 276 in 2025, and 271 in 2026.

Screening and thematic classification were performed by the author. Titles and abstracts were reviewed, and full texts were consulted when the principal research focus could not be determined from the title and abstract alone. The thematic categories were not predefined before the search. Rather, recurring research themes were identified during review of the eligible literature and were iteratively consolidated into 13 categories (Table 1). When a study addressed more than one domain, it was assigned to the single category judged to best represent its primary research objective.

No second independent reviewer or formal assessment of inter-rater agreement was used. Accordingly, the thematic classification inevitably involved a degree of author judgment and should be regarded as descriptive rather than as a formally validated bibliometric classification.

This literature-mapping exercise was not intended to constitute a systematic or scoping review, and no PRISMA-based study selection procedure, formal risk-of-bias assessment, or bibliometric analysis was performed. Its purpose was to provide contextual information on recent research themes relevant to the Four Kai framework. Therefore, the resulting article counts should be interpreted as descriptive indicators of research patterns rather than as quantitative estimates of the relative importance, strength of evidence, or research activity of individual fields. Because the categories were developed inductively and differed in conceptual breadth, direct quantitative comparisons among categories should also be interpreted cautiously.

Representative studies discussed in the subsequent narrative sections were selected primarily for their relevance to pediatric and adolescent populations and to the conceptual questions addressed in this review. Their selection was not based on a formal systematic study selection procedure or standardized methodological quality assessment.

### 2.2. Descriptive Research Trends

Table 2 shows the distribution of the 13 thematic categories over the five-year period. Overall, the largest category within this descriptive classification was Integrated lifestyle (268 studies), followed by Physical activity/Exercise (144 studies), Sleep health (139 studies), Sleep disorders (138 studies), Family/Parenting/Environment (110 studies), and Diet/Nutrition (104 studies). Sleep disorders showed the highest annual count in 2026 (48 studies). Whether this increase represents a genuine shift in research activity or incomplete indexing of recent publications cannot be determined from the present analysis.

This broader perspective is exemplified by the concept of 24-h movement behaviors, which recognizes sleep, physical activity, and sedentary behavior as interdependent behaviors competing for the limited time available within a 24-h day rather than as independent lifestyle factors. Recent systematic reviews and meta-analyses have shown that adherence to the 24-h movement guidelines is associated with better physical health, mental health, cognitive performance, academic achievement, and health-related quality of life in children and adolescents [8,12,13,14,15]. Furthermore, an increasing number of intervention studies have attempted to improve sleep, physical activity, and sedentary behavior simultaneously, highlighting the importance of addressing multiple lifestyle behaviors in an integrated manner [16].

More recently, this integrated perspective has expanded beyond movement behaviors to include dietary behaviors. Studies have increasingly examined how dietary quality, breakfast consumption, meal timing, meal regularity, and physical activity are associated with sleep duration, sleep quality, sleep regularity, and other dimensions of sleep health [8,17,18,19]. Likewise, sleep health is now recognized as a multidimensional construct encompassing not only sleep duration but also regularity, timing, satisfaction, and daytime alertness. Moreover, children’s sleep is increasingly understood to be influenced not only by their own behaviors but also by parental practices, family routines, school environments, neighborhood characteristics, socioeconomic conditions, and environmental exposures. Consequently, recent studies have evaluated interventions targeting both parents and children to improve sleep, diet, and physical activity simultaneously, while others have examined associations between sleep and family stress, neighborhood environments, and environmental exposures [20,21,22,23]. Accordingly, studies classified as Integrated lifestyle in the present review included investigations combining sleep with diet and physical activity, studies based on the 24-h movement framework, research addressing multiple health behaviors including screen use, parental intervention studies, and investigations examining family, school, and community environments.

Despite these advances, an important gap remains. The Gut–brain axis category, which included studies addressing gastrointestinal symptoms, constipation, gut microbiota, digestive function, and gut–brain interactions, comprised 45 of the 1196 publications across the five-year period. Moreover, even recent research on the gut–brain axis has focused primarily on the gut microbiome, inflammation, metabolism, abdominal pain, irritable bowel syndrome, and functional constipation, whereas very few studies have investigated everyday bowel habits, such as bowel movement frequency or bowel regularity in healthy children, together with sleep and other lifestyle behaviors [24,25]. Thus, bowel habits were rarely represented as an independent research theme and were generally examined within studies of gastrointestinal disorders rather than as part of everyday physiological functioning.

This relative underrepresentation of bowel habits should not be interpreted as evidence that bowel function is necessarily equivalent in importance to sleep, eating, or physical activity within an integrated lifestyle model. Rather, it identifies an area in which the available evidence remains comparatively limited. This gap provides the rationale for examining bowel habits alongside sleep, eating, and physical activity within the hypothesis-generating Four Kai framework developed in the following sections.

## 3. Bowel Habits and Sleep

In the present review, bowel habits refer primarily to everyday physiological characteristics such as bowel movement frequency, defecation regularity, stool characteristics, and comfortable defecation in otherwise healthy children, rather than to gastrointestinal diseases. Among the four physiological behaviors considered in the present review, bowel habits have received the least attention in pediatric sleep research. Despite the growing recognition of integrated lifestyle behaviors, bowel function is still rarely evaluated together with sleep, diet, physical activity, and other daily behaviors. Even the Japanese Sleep Guidelines for Health Promotion 2023 published by the Ministry of Health, Labour and Welfare do not identify bowel habits as a major component of sleep hygiene [26].

Direct pediatric evidence examining everyday bowel function together with sleep and other daily behaviors remains scarce. Consequently, much of the available evidence discussed below derives from studies of constipation, disorders of gut–brain interaction (DGBIs), irritable bowel syndrome, abdominal pain, or other gastrointestinal conditions. Such studies provide indirect background for considering possible interactions between sleep and bowel function but should not be interpreted as direct evidence for the role of healthy everyday bowel habits within the Four Kai framework. This section therefore summarizes the available evidence while highlighting this important gap in the literature.

As indirect supporting evidence, studies in adults have linked constipation with poor sleep quality, sleep deficiency, and abnormal sleep duration [27,28]. A recent systematic review and meta-analysis supported an overall association between sleep disorders and constipation [29], while several population-based studies reported U-shaped relationships, with both short and long sleep duration associated with a higher prevalence of constipation or abnormal bowel symptoms [30,31,32], similar to associations reported for cognitive decline [33], biological aging [34], BMI [35], and mortality [36]. In addition, a large-scale longitudinal cohort study showed that sleep patterns were prospectively associated with the risk of digestive diseases, providing further support for a temporal relationship between sleep and gastrointestinal health [37]. Constipation has also been recognized as a relevant non-motor feature in REM sleep behavior disorder and related neurodegenerative conditions [38,39]. However, because much of this evidence is derived from adult or clinical populations, it should be regarded only as supportive physiological background. Its relevance to everyday bowel function in healthy children and adolescents remains uncertain and should be interpreted cautiously.

From the conceptual framework adopted in the present review, several studies have examined the relationships between bowel habits, diet, and physical activity. Chien et al. reported that infrequent bowel movements among Taiwanese adolescents were associated with insufficient water and dietary fiber intake as well as prolonged sedentary behavior [40]. Similarly, Huang et al., in a study of 33,692 secondary school students in Hong Kong, found that constipation was associated with insufficient exercise, lower levels of habitual light physical activity, and prolonged sedentary behavior, with the likelihood of constipation increasing as inactivity-related factors accumulated [41]. These findings suggest that bowel function is influenced not only by dietary fiber and water intake but also by habitual physical activity. Driessen et al. prospectively followed 347 preschool children and objectively measured physical activity using accelerometers. They found that children with higher levels of physical activity were less likely to develop functional constipation at four years of age, although the strength of the association varied according to age and the timing of assessment [42]. This study is noteworthy because it used objective measurements rather than questionnaire-based estimates and examined the temporal relationship between physical activity and subsequent constipation. More recently, investigators have begun to examine bowel habits from the perspective of 24-h movement behaviors. Komeno et al. evaluated physical activity and bowel habits in children aged 4–6 years using accelerometry and demonstrated that daily step counts and moderate-to-vigorous physical activity were associated with healthier bowel habits. Moreover, replacing part of sedentary time or light-intensity activity with moderate-to-vigorous physical activity was also associated with improved bowel function [43]. This study extends the contemporary concept that sleep, sedentary behavior, light physical activity, and moderate-to-vigorous physical activity compete for time within a 24-h day to the field of bowel health. Recent systematic reviews have likewise examined associations between physical inactivity, sedentary behavior, and constipation in children. However, considerable heterogeneity exists in the definitions of both physical activity and constipation, and most available studies are cross-sectional. Consequently, it remains unclear whether insufficient physical activity contributes to constipation or whether children with constipation become less physically active [44].

The relationship between sleep and gastrointestinal function has also been increasingly investigated within the framework of DGBI, which includes functional constipation, irritable bowel syndrome, functional abdominal pain, and functional dyspepsia. A systematic review by Robbertz et al. demonstrated that children with DGBI frequently experience sleep problems and that poorer sleep is associated with abdominal pain, psychological symptoms, and impaired daily functioning [45]. Sleep deprivation and sleep fragmentation may exacerbate gastrointestinal symptoms through altered pain perception, autonomic dysregulation, inflammatory processes, and emotional dysregulation, whereas abdominal pain and constipation may themselves interfere with sleep initiation and maintenance. These findings support a bidirectional relationship between sleep and gastrointestinal symptoms. However, most DGBI studies focus on gastrointestinal disorders rather than everyday bowel habits in otherwise healthy children and therefore differ conceptually from the perspective adopted in the present review.

Among studies simultaneously evaluating sleep, physical activity, and bowel function, the work of Piriyakitphaiboon et al. deserves particular attention. Their study investigated the relationships among screen use, physical activity, sleep duration, and bladder and bowel dysfunction in Thai children [46]. Although this study is important because it assessed sleep and physical activity together with elimination function, its primary outcome was bladder and bowel dysfunction, including urinary symptoms, rather than bowel movement frequency or bowel regularity. Furthermore, dietary behaviors and daytime functioning—defined in the present review as cognitive performance, academic achievement, emotional regulation, alertness, social functioning, and health-related quality of life—were not evaluated.

In Japan, several studies based on the Toyama Birth Cohort have examined bowel habits in relation to a wide range of lifestyle behaviors. Yamada et al. analyzed 7762 children aged 9–10 years and found that infrequent bowel movements and irregular defecation timing were associated with breakfast skipping, lower physical activity, television viewing, and sleep schedules [47]. Rather than considering constipation solely as a gastrointestinal disorder, this study positioned bowel habits within the broader context of daily lifestyle, including sleep, diet, physical activity, and screen behaviors. Their subsequent study involving 7998 children further demonstrated that constipation was associated not only with dietary habits but also with psychological stress, irritability, reluctance to attend school, and parent–child interactions [48]. These findings suggest that bowel habits are closely related to both physical lifestyle factors and the psychosocial environment.

A nationwide survey conducted by the Japan Toilet Labo in 2025 collected seven-day bowel diaries from 11,771 elementary and junior high school students [49]. Approximately one-quarter of elementary school children were considered to have constipation or a tendency toward constipation based on bowel movement frequency and stool consistency according to the Bristol Stool Form Scale [50]. Furthermore, children who did not consume breakfast every day were more likely to have infrequent bowel movements than those who ate breakfast daily. These findings further support the view that bowel habits should be considered not merely as gastrointestinal symptoms but as part of everyday lifestyle behaviors.

In our own survey of 2722 Japanese students from the fifth grade of elementary school through the third year of high school, poor bowel habits were associated with breakfast skipping, lower physical activity, longer weekday screen time, and poorer academic performance, although the associated factors differed somewhat across school stages [51]. Although no independent association between bowel habits and sleep was identified in the multivariable analysis, subsequent analyses according to bowel movement frequency demonstrated that students with bowel movements occurring no more than twice per week had significantly later bedtimes and shorter weekday sleep duration than those with daily bowel movements [52]. These findings suggest that infrequent bowel movements may be associated with delayed sleep schedules and insufficient sleep duration. Similarly, a recent population-based study of Japanese elementary school children showed that constipation was independently associated with insufficient and non-restorative sleep [53].

It should be emphasized, however, that bowel habits currently represent the least empirically supported component of the Four Kai framework. Much of the available pediatric evidence derives from children with functional constipation or other gastrointestinal disorders, whereas evidence concerning everyday bowel function in otherwise healthy children remains limited. Furthermore, existing studies do not establish that normal bowel function contributes to sleep health or daytime functioning to the same extent as sleep, eating, or physical activity. Bowel habits should therefore be regarded at present as a promising and hypothesis-generating component of the Four Kai framework rather than as a confirmed pillar with an evidence base equivalent to that of the other three components. Future studies evaluating bowel movement frequency, regularity, stool consistency, sleep, and daytime functioning simultaneously in healthy pediatric populations will be necessary to determine its role within the framework.

## 4. Diet and Sleep

### 4.1. Dietary Patterns, Breakfast, and Sleep

The relationship between breakfast and sleep has been extensively investigated in pediatric populations. In addition to its association with sleep, breakfast consumption has consistently been linked with better cognitive performance, healthier dietary quality, and more favorable cardiometabolic outcomes [54,55,56]. More broadly, recent research has increasingly examined dietary quality and eating behaviors in relation to sleep rather than considering individual nutrients or foods in isolation.

In a systematic review of dietary habits and sleep among children and adolescents, Alibabaei et al. reported that healthy dietary patterns were generally associated with longer sleep duration and better sleep quality, whereas diets high in fat and sugar, irregular eating behaviors, and nighttime eating were associated with shorter sleep duration and poorer sleep quality [54]. However, substantial heterogeneity existed among studies with respect to participant characteristics and the assessment of both diet and sleep, limiting causal interpretation.

Importantly, the relationship between sleep and diet should not be regarded as unidirectional. A systematic review specifically examining bidirectional associations between sleep and dietary intake or eating behaviors in children found evidence for associations in both directions, although most of the included studies were cross-sectional and therefore could not establish temporality [57]. Thus, the relationship between diet and sleep is likely to be bidirectional: dietary quality and eating behaviors may influence sleep, while insufficient or poor-quality sleep may in turn influence dietary intake and eating behaviors. However, the relative contribution and causal direction of these pathways remain uncertain, and shared behavioral, environmental, and biological factors may also contribute to the observed associations.

### 4.2. Meal Timing, Regularity, and Chrono-Nutrition

Beyond dietary composition, the temporal organization of eating has become an increasingly important focus of research. The concept of chrono-nutrition emphasizes not only what people eat but also when they eat. Recent reviews have suggested that nutrition, physical activity, and sleep should be viewed as interacting lifestyle behaviors rather than independent determinants of health [58]. Chrono-nutrition research has further highlighted that meal timing, meal regularity, and the temporal distribution of energy intake are associated with sleep, circadian rhythms, and metabolic health [59,60]. Consequently, dinner timing, nighttime eating, and meal regularity have become increasingly relevant to pediatric sleep research [17,61].

In a systematic review involving 46 studies and approximately 210,000 participants, Dutil et al. reported that children and adolescents with later bedtimes had not only shorter sleep duration and poorer sleep quality but also less healthy dietary habits, lower levels of physical activity, and greater sedentary behavior [61]. Spaeth et al. further demonstrated associations among sleep duration, meal timing, and energy balance in school-aged children [62]. Taken together, these studies suggest that the relationship between sleep and eating extends beyond dietary composition and involves the temporal organization of eating, sleep, physical activity, and sedentary behavior. However, the predominantly observational nature of the evidence precludes conclusions about causal direction.

We previously classified dinner habits into three groups—regular early dinner, regular but late dinner, and irregular dinner—and examined their associations with lifestyle behaviors [63]. Children and adolescents with a regular early dinner had earlier bedtimes, longer sleep duration, less daytime sleepiness, and better self-reported academic performance, whereas those with a regular but late dinner had later bedtimes, shorter sleep duration, longer weekday screen time, greater daytime sleepiness, and poorer academic performance. Participants with irregular dinner habits spent the longest time in after-school activities. Moreover, the proportion of adolescents with a regular but late dinner increased with age, reaching 41.1% among high school students. These findings are consistent with the broader chrono-nutrition literature and suggest that delayed or irregular dinner timing may occur as part of a broader pattern of altered daily behavioral rhythms rather than as an isolated dietary behavior [17,61].

### 4.3. Integrated Evidence and Future Directions

More recently, sleep has increasingly been evaluated as the multidimensional construct of sleep health rather than solely in terms of sleep duration. Ballester-Navarro et al. investigated associations among sleep health, dietary habits, and physical activity in 373 adolescents and found that later bedtimes and midsleep times were associated with more frequent snacking, greater energy intake, and longer sedentary time, while poorer sleep regularity was associated with increased sedentary behavior [17]. Although dinner timing was not directly assessed, this study illustrates the value of examining multiple dimensions of sleep together with dietary and activity behaviors.

Intervention evidence remains limited. Matsumoto et al. reported that improving breakfast quality by adding granola was accompanied by favorable changes in nutritional balance, sleep, and bowel habits [64]. Although the study was relatively small, it provides preliminary evidence that modification of one daily behavior may be associated with changes in others.

Overall, three main patterns emerge from the available evidence. First, sleep and eating appear to be bidirectionally associated, involving not only dietary quality but also meal timing and regularity, although the causal pathways remain incompletely established. Second, these associations frequently occur alongside differences in physical activity, sedentary behavior, screen use, and other aspects of daily behavioral timing, suggesting that sleep–diet relationships should be interpreted within the broader organization of the 24-h day. Third, most evidence remains observational, and relatively few pediatric studies have directly examined dinner timing or tested whether modifying eating behavior produces changes in sleep or other physiological behaviors. Thus, the available evidence supports multiple associations among sleep, eating, and activity-related behaviors but does not establish that they operate as a single integrated physiological system.

Future studies should therefore examine meal timing and regularity together with multidimensional sleep health, physical activity, and daytime functioning, using longitudinal and intervention designs capable of addressing temporal and potentially bidirectional relationships. Everyday bowel habits should also be assessed where appropriate, because their relationship with sleep and eating remains substantially less studied. Such studies would help determine whether the associations currently observed among individual or paired behaviors extend to the broader functional integration proposed by the Four Kai framework.

## 5. Physical Activity and Sleep

Physical activity is widely recognized as an important determinant not only of physical health but also of sleep, mental health, cognitive function, and academic performance in children and adolescents. Recent reviews have emphasized that physical activity and sleep should be considered together because both are associated with cognitive function and overall health, although their causal and reciprocal relationships remain incompletely understood [58,65].

Systematic reviews and meta-analyses have consistently demonstrated that regular physical activity is generally associated with better sleep outcomes, although the magnitude and consistency of these associations vary across studies according to the type, intensity, and amount of physical activity [66,67,68]. Importantly, the relationship between physical activity and sleep may operate in both directions. In an actigraphy-based study of 417 adolescents, Master et al. examined daily temporal associations in both directions and found that greater moderate-to-vigorous physical activity during the day was associated with earlier sleep onset, longer sleep duration, and higher sleep maintenance efficiency that night. Conversely, nighttime sleep characteristics were also associated with physical activity and sedentary behavior on the following day, although these associations were complex and did not consistently indicate that better or longer sleep resulted in greater next-day physical activity [69]. Thus, current pediatric evidence supports the possibility of bidirectional interactions between physical activity and sleep, but their direction, magnitude, and temporal characteristics remain incompletely established.

Furthermore, a recent network meta-analysis in adults identified a dose–response relationship between exercise and sleep quality, suggesting that an optimal rather than maximal amount of exercise may provide the greatest benefit [70]. Although this analysis was conducted primarily in adults, it provides useful physiological background for considering whether the amount of physical activity may also be relevant to sleep in pediatric populations. However, these adult findings should not be interpreted as direct evidence for an optimal exercise dose in children and adolescents.

Observational studies in pediatric populations also suggest that the frequency of physical activity may be associated with daytime functioning. In our previous study involving Japanese students from the fifth grade of elementary school through the third year of high school, more frequent participation in physical activity was associated with earlier wake-up times, less frequent breakfast skipping, and shorter weekend screen time, indicating an overall healthier lifestyle [71]. Interestingly, however, the relationship between physical activity frequency and daytime sleepiness was U-shaped rather than linear. Increased daytime sleepiness was observed not only among students who did not exercise at all but also among those who exercised every day, whereas the lowest level of daytime sleepiness was found among students exercising approximately one to two days per week. This observational association does not demonstrate that daily or high-frequency physical activity itself causes daytime sleepiness, because unmeasured factors such as exercise intensity, duration, training schedules, sleep opportunity, or other lifestyle characteristics may have contributed to the observed pattern. Accordingly, these findings should be regarded as generating a hypothesis regarding the amount and pattern of physical activity rather than as evidence for an established optimal or excessive level of physical activity in children and adolescents.

More recently, physical activity has increasingly been examined not as an isolated health behavior but as one component of an integrated lifestyle. Ballester-Navarro et al. simultaneously evaluated sleep health, dietary behaviors, and physical activity in adolescents and demonstrated that later bedtimes and later midsleep times were associated with more frequent snacking, greater total energy intake, and longer sedentary time [17]. Although the study did not directly examine exercise interventions, it highlighted the value of considering physical activity, sleep, and dietary behaviors together within the organization of the 24-h day. Additional evidence suggests that sedentary behavior is associated with sleep disturbance, potentially involving inflammatory pathways [72], further supporting the importance of considering physical activity and sedentary time together when examining sleep health.

Taken together, the available evidence suggests a potentially bidirectional relationship between physical activity and sleep and indicates that both behaviors are associated with other aspects of daily functioning, including sedentary behavior, dietary habits, and daytime alertness. However, most of these relationships have been examined individually or in limited combinations, and current evidence does not establish that sleep, eating, bowel habits, and physical activity operate as a single integrated physiological system.

Within the Four Kai framework, the potential reciprocal relationship between sleep and physical activity provides one example of how daily physiological behaviors may influence one another. Physical activity is also particularly relevant to the proposed concept of adaptive pleasure because appropriately matched activity may be accompanied by subjective experiences of refreshment, accomplishment, or well-being. Such experiences could potentially contribute to behavioral repetition and positive self-regulation. However, whether adaptive pleasure actually mediates the relationships among physical activity, subsequent behavior, and sleep has not been directly tested and should therefore be regarded as a hypothesis for future research.

Thus, the evidence reviewed in this section supports associations between physical activity, sleep, and other daily behaviors, while the Four Kai framework extends these observations by proposing testable questions regarding their possible functional integration. This perspective provides the foundation for the following discussion of potential neurobiological mechanisms underlying adaptive pleasure and positive self-regulation.

## 6. Neurobiology of the Four Kai and Positive Self-Regulation

Before considering the potential neurobiological mechanisms of the Four Kai framework, an important distinction should be emphasized. Existing evidence supports numerous individual and pairwise associations among sleep, eating, physical activity, bowel function, and health outcomes. However, these findings do not establish that all four behaviors operate as a single integrated physiological system. Likewise, no study has directly demonstrated the proposed sequence linking the Four Kai to adaptive pleasure, positive self-regulation, and daytime functioning. The neurobiological mechanisms discussed below should therefore be interpreted as a hypothesis-generating synthesis of findings from related research fields rather than as evidence for an established causal pathway. Accordingly, the following section first summarizes relevant established neurobiological findings and then considers how these findings might inform the proposed Four Kai framework; the latter should be understood as a conceptual interpretation rather than as evidence derived directly from studies of the Four Kai.

Within the proposed Four Kai framework, sleep, eating, bowel habits, and physical activity are considered four fundamental physiological behaviors that may be accompanied by distinct forms of adaptive pleasure. Here, adaptive pleasure is conceptualized within the framework as a potential subjective signal associated with the restoration or maintenance of homeostasis. Such pleasurable experiences are hypothesized to contribute to the repetition of health-promoting behaviors and thereby may support positive self-regulation.

Recent advances in neuroscience indicate that pleasure is not generated by a single brain region or neurotransmitter but rather emerges from coordinated interactions among neural systems involved in reward processing, emotion, interoception, and cognitive control [73,74,75]. The mesolimbic dopaminergic pathway, extending from the ventral tegmental area to the nucleus accumbens, plays an important role in reward anticipation and motivational drive (“wanting”). In contrast, the hedonic experience of pleasure (“liking”) involves more localized neural processes, including hedonic hotspots within the nucleus accumbens and ventral pallidum, together with cortical regions such as the orbitofrontal cortex (OFC), and cannot be explained solely by dopamine [73,74,75,76]. Reward-related information is also processed within prefrontal regions involved in value-based decision making and behavioral regulation. The ventromedial PFC and OFC contribute to valuation and outcome evaluation, whereas the dorsolateral prefrontal cortex (PFC) contributes to goal-directed behavior and cognitive control. In addition, the anterior cingulate cortex (ACC) is involved in conflict monitoring and prediction-related processing, while the anterior insula plays an important role in integrating interoceptive signals related to internal bodily states [77,78,79,80]. Together, these systems provide a general neurobiological background for understanding how physiological states, subjective experience, valuation, and subsequent behavioral choices may interact.

However, the evidence underlying this neurobiological background is heterogeneous and derives from animal studies, experimental and neuroimaging studies in humans, and observational research, with much of the mechanistic evidence obtained outside pediatric populations. Extrapolation across species, experimental paradigms, age groups, and levels of analysis should therefore be made cautiously, and these findings should not be interpreted as direct evidence for the proposed mechanisms of the Four Kai in children and adolescents.

Within the proposed Four Kai framework, these established neurobiological functions are hypothesized to be relevant to the subjective experiences associated with the appropriate fulfillment of different physiological needs. Adequate sleep may be followed by feelings of restoration and refreshment; eating may produce satisfaction and comfortable satiety as energy needs are met; bowel evacuation may be accompanied by relief as interoceptive discomfort resolves; and physical activity may be associated with enjoyment, vitality, refreshment, or a sense of achievement. These experiences arise from different physiological processes and should not be assumed to share an identical neural mechanism. The Four Kai framework proposes that they may nevertheless share the broader functional feature of signaling, at a subjective level, the restoration or maintenance of a physiologically favorable state.

Drawing on established concepts of self-regulation [3], self-regulation is used in the present review to refer to the processes through which individuals monitor and adjust their behavior, internal states, and responses in ways that support the attainment or maintenance of desired physiological and behavioral states. Positive self-regulation refers more specifically to the hypothesized process by which adaptive subjective experiences associated with appropriately fulfilled physiological needs may contribute to the repetition and maintenance of health-promoting behaviors. The term “positive” is used here to emphasize reinforcement through adaptive subjective experience rather than regulation driven primarily by avoidance, restriction, or correction of unhealthy behavior.

From this perspective, adaptive pleasure is hypothesized not only as a possible outcome of appropriate physiological behaviors but also as a potential contributor to their repetition. Adequate sleep, appropriate eating, healthy bowel function, and appropriate physical activity may be accompanied by adaptive pleasurable experiences, which could in turn influence motivation to repeat these behaviors. Repeated associations between appropriate physiological behaviors and such subjective experiences might thereby contribute to self-regulation and the development or maintenance of healthy behavioral patterns. Importantly, this proposed sequence has not been directly demonstrated and is intended as a hypothesis-generating conceptual framework rather than as an established neurobiological mechanism.

The distinction between adaptive and non-adaptive pleasure is also important. Strong hedonic or motivational responses do not necessarily promote physiological regulation or healthy behavior. In addictive or compulsive behaviors, including substance use, gambling, overeating, and excessive social media use, reward-related processes may become disproportionately oriented toward motivational drive (“wanting”) relative to hedonic experience (“liking”), while processes involved in behavioral control and value-based decision making may also be altered [73,74]. These observations illustrate more generally why pleasure itself should not be equated with adaptive pleasure in the Four Kai framework.

Taken together, contemporary neuroscience provides a plausible biological context in which homeostatic regulation, reward processing, interoception, valuation, and self-regulation interact. However, this general neurobiological evidence should not be interpreted as direct support for the specific Four Kai pathway. Although evidence supports numerous individual associations involving sleep, eating, bowel function, physical activity, and daytime functioning, direct empirical evidence for a common pathway linking these behaviors through adaptive pleasure and positive self-regulation to improved daytime functioning is currently lacking.

Accordingly, the Four Kai framework should be viewed as a hypothesis-generating conceptual framework rather than as an established biological model. It does not imply that the four physiological behaviors share identical neural mechanisms. Rather, it proposes that partially overlapping systems involved in homeostatic regulation, interoception, reward processing, valuation, and cognitive control may provide candidate mechanisms relevant to the hypothesized functional interrelationships among these behaviors. Future studies should determine whether adaptive pleasure can be operationalized and empirically demonstrated as a common or partially shared process linking these daily physiological behaviors.

## 7. Toward Better Daytime Functioning in Children and Adolescents

**Figure 1:**
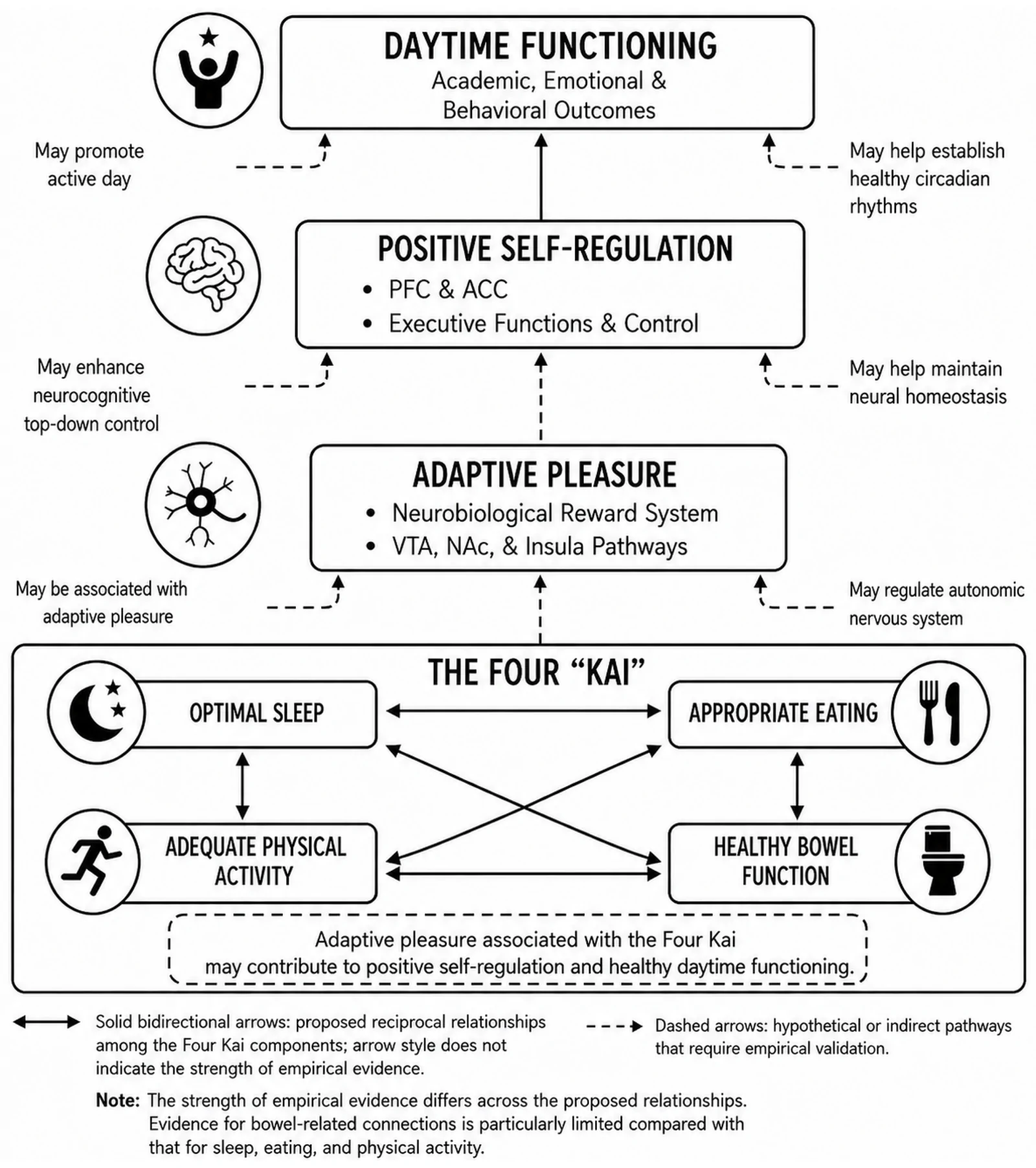
Proposed conceptual framework of the Four Kai and positive self-regulation.

The present review does not argue that the Four Kai framework has been empirically validated. Rather, it proposes a conceptual framework that integrates independent lines of evidence concerning sleep, eating, bowel habits, physical activity, adaptive pleasure, self-regulation, and daytime functioning. The proposed Four Kai framework is summarized in Figure 1.

This diagram illustrates the proposed relationships among the four components of the Four Kai—Optimal Sleep, Pleasurable Eating, Adequate Physical Activity, and Appropriate Bowel Function—and their hypothesized links with adaptive pleasure, positive self-regulation, and daytime functioning. Solid bidirectional arrows among the four physiological behaviors depict their proposed reciprocal relationships and the structural organization of the framework; they should not be interpreted as indicating equivalent levels of empirical support. Although relationships among sleep, eating, and physical activity are supported by substantial evidence, the strength of evidence for specific reciprocal relationships varies, and evidence regarding bowel habits remains comparatively limited. Accordingly, connections involving bowel function should be regarded as preliminary and hypothesis-generating. Within the proposed framework, appropriate fulfillment of the Four Kai may be associated with adaptive pleasure, potentially involving neural systems related to reward processing and interoception, including the ventral tegmental area (VTA), nucleus accumbens (NAc), and insula. Adaptive pleasure is, in turn, hypothesized to contribute to positive self-regulation, potentially involving prefrontal and anterior cingulate processes. Positive self-regulation may support better daytime functioning, which may in turn help sustain healthy behavioral rhythms. Dashed arrows indicate hypothetical or indirect pathways that require empirical validation. Thus, the figure represents a hypothesis-generating conceptual framework rather than an established causal pathway.

As illustrated, the framework integrates four daily physiological behaviors through the concepts of adaptive pleasure and positive self-regulation to explain their potential contributions to healthy daytime functioning. Importantly, however, the evidence supporting each component is not equally strong. While substantial evidence supports associations among sleep, eating, and physical activity, evidence regarding bowel habits remains comparatively limited. Accordingly, the proposed role of bowel habits should currently be regarded as a preliminary component of this conceptual framework and an important priority for future research. In the conceptual framework presented in this review, adaptive pleasure is viewed not only as an outcome of healthy behaviors but also as a potential factor that helps sustain them. Adequate sleep, healthy eating habits, regular bowel function, and appropriate physical activity may each be accompanied by distinct forms of adaptive pleasure. These pleasurable experiences may encourage the repetition of healthy behaviors, reinforce self-regulation, and contribute to a positive feedback cycle, referred to here as positive self-regulation, which may ultimately support healthy daytime functioning. At present, however, no study has directly demonstrated this entire sequence of events, and the proposed pathway should therefore be regarded as a conceptual hypothesis for integrating existing evidence rather than as an established biological mechanism.

Substantial evidence supporting the individual components of this framework has accumulated. Recent systematic reviews and meta-analyses have consistently demonstrated that adequate sleep is associated with reduced daytime sleepiness, better cognitive performance, improved emotional regulation, enhanced psychological well-being, and superior academic achievement [81,82]. Likewise, systematic reviews by Hoyland et al. [83] and Adolphus et al. [84] have shown that breakfast consumption is associated with improvements in attention, executive function, memory, and academic performance. Appropriate dietary habits, particularly when combined with regular physical activity, have also been associated with improved morning cognitive performance, mathematical achievement, and reaction time [54,55]. Similarly, regular physical activity has been shown to improve cognitive function, executive function, attention, emotional regulation, and academic performance, although the magnitude of these benefits varies according to the type, frequency, duration, and intensity of exercise [85,86,87].

Evidence directly linking bowel habits with cognitive performance or academic achievement remains limited. Nevertheless, functional constipation has consistently been associated with poorer health-related quality of life, psychological well-being, school functioning, and daily activities in children [45,88,89]. Furthermore, treatment of constipation has been reported to improve quality of life, suggesting that healthy bowel function may also contribute to the maintenance of daytime functioning [89]. However, because much of this evidence derives from children with constipation or other gastrointestinal problems rather than healthy pediatric populations, it should be regarded as indirect support for the proposed role of everyday bowel function within the Four Kai framework.

Taken together, these findings indicate that sleep, eating, bowel habits, and physical activity are each associated with different aspects of daytime functioning. Importantly, however, previous studies have generally examined these physiological behaviors individually or in limited combinations. None has directly evaluated adaptive pleasure, self-regulation, and daytime functioning as components of a single integrated pathway. Nevertheless, the available evidence is consistent with the possibility that adequate sleep supports alertness and cognition, healthy eating provides the energy required for cognitive performance, healthy bowel function may reduce physical discomfort and limitations in daily life, and appropriate physical activity promotes cognitive and emotional functioning.

Viewed collectively, these findings suggest that the Four Kai framework, together with the concept of positive self-regulation, may provide a useful conceptual basis for integrating these otherwise largely independent observations.

Although the Four Kai framework has not yet been empirically validated as an integrated intervention framework, it may offer a practical structure for considering children’s daily physiological behaviors more comprehensively. In clinical practice, assessment of sleep problems could be accompanied by brief screening of meal timing and regularity, bowel habits, and physical activity, rather than focusing on sleep in isolation. In schools, health education could similarly encourage children and families to recognize potential relationships among sleep, regular meals, comfortable bowel habits, physical activity, and daytime alertness and well-being. At the public health level, the framework may provide a simple organizing concept for health-promotion messages that address multiple daily behaviors together rather than as separate recommendations. Importantly, these applications should currently be regarded as potential uses of the framework rather than as evidence-based Four Kai interventions, and their feasibility and effectiveness require empirical evaluation.

Empirical validation of the Four Kai framework will require several complementary approaches. First, longitudinal observational studies should simultaneously assess sleep, eating, bowel habits, physical activity, adaptive pleasure, self-regulation, and daytime functioning to determine their temporal relationships and whether changes in one domain predict subsequent changes in others. Standardized assessments should include sleep duration, timing, regularity, and quality; meal timing and regularity; bowel movement frequency, regularity, and stool characteristics; and the amount and timing of physical activity. Wearable devices and smartphone-based diaries may facilitate repeated longitudinal assessment of these behaviors. Second, adaptive pleasure should be operationalized using behavior-specific subjective measures of restoration, refreshment, satisfaction, relief, or well-being, and its relationship with subsequent behavioral repetition and self-regulation should be tested. Third, prospective intervention studies could compare interventions targeting multiple Four Kai behaviors with conventional single-behavior approaches to determine whether integrated interventions produce greater improvements in self-regulation and daytime functioning. Such studies would also allow formal testing of whether adaptive pleasure and self-regulation mediate any observed effects. These approaches could determine which proposed relationships are supported, which require modification, and whether the Four Kai framework provides added explanatory or practical value beyond existing lifestyle frameworks.

## 8. Conclusions

### 8.1. What Is Already Established

The evidence reviewed in this article supports multiple associations involving sleep, eating, physical activity, bowel function, and daytime functioning in children and adolescents. However, the strength and extent of evidence differ substantially across these domains, with considerably less evidence available regarding everyday bowel habits in otherwise healthy children.

### 8.2. What Remains Hypothetical

Whether sleep, eating, bowel habits, and physical activity operate together as a single integrated physiological system remains to be established. Bowel habits, in particular, should currently be regarded as a promising and hypothesis-generating component of the Four Kai framework rather than as a component supported by an evidence base equivalent to that of sleep, eating, or physical activity. The Four Kai framework presented in this review is therefore best regarded as a hypothesis-generating conceptual framework for integrating existing evidence rather than as an evidence-based causal model. Viewing these physiological behaviors through the perspectives of adaptive pleasure and positive self-regulation may provide a useful way of generating testable hypotheses across research fields that have traditionally been investigated separately. The framework is not intended to replace existing concepts such as sleep health or integrated lifestyle behaviors but rather to offer a complementary perspective emphasizing the possibility of functional interactions among daily physiological behaviors. At present, however, direct empirical evidence linking the four behaviors, adaptive pleasure, positive self-regulation, and daytime functioning within a single integrated pathway remains lacking.

### 8.3. Limitations

Several limitations should be considered when interpreting this review. First, the literature-mapping component was descriptive rather than systematic, scoping, or bibliometric and was limited to PubMed. No formal PRISMA-based study selection procedure or risk-of-bias assessment was performed. In addition, all screening and thematic classification were performed by a single author, without independent classification or assessment of inter-rater agreement. The thematic categories were developed inductively and differed in conceptual breadth; consequently, their publication counts should be regarded only as descriptive indicators of the research themes identified by this search strategy and not as quantitative measures of the relative importance, research activity, or strength of evidence of individual fields.

Second, the PubMed search was restricted to publications containing “sleep” in the title, increasing specificity but potentially excluding relevant studies in which sleep was reported only in the abstract or as a secondary outcome. No language restriction was applied. The descriptive mapping was also restricted to publications from January through June of each year from 2022 to 2026 to permit comparison across equivalent six-month periods because only the first half of 2026 was available at the time of the search. Consequently, it does not represent the complete literature published during these calendar years, and the 2026 findings may additionally have been affected by incomplete indexing at the time of the search.

Third, representative studies discussed in the narrative sections were selected primarily for their relevance to pediatric and adolescent populations and to the conceptual questions addressed in this review rather than through a formal systematic selection procedure. In addition, some of the studies used to illustrate relationships within the Four Kai framework were conducted by the author. Although these studies provide relevant pediatric data, their inclusion may increase the influence of the author’s own research perspective on the conceptual synthesis.

Fourth, the empirical evidence supporting the four components of the framework is uneven. Evidence concerning everyday bowel habits in otherwise healthy children and adolescents remains particularly limited, and much of the available evidence derives from adults or pediatric populations with constipation or other gastrointestinal disorders. The neurobiological interpretation likewise draws on heterogeneous evidence from animal studies, human experimental and neuroimaging research, and observational studies, much of which was not conducted specifically in pediatric populations. Extrapolation across species, developmental stages, experimental paradigms, and levels of analysis should therefore be made cautiously.

Finally, the Four Kai framework itself has not been empirically validated as an integrated physiological model. In particular, no study has directly demonstrated the proposed pathway linking sleep, eating, bowel habits, and physical activity through adaptive pleasure and positive self-regulation to daytime functioning. These relationships should therefore be regarded as hypothesis-generating propositions that require testing in prospective longitudinal and intervention studies.

### 8.4. Future Research

Future longitudinal and intervention studies should simultaneously evaluate sleep, eating, bowel habits, physical activity, adaptive pleasure, self-regulation, and daytime functioning to test whether the relationships proposed in the Four Kai framework can be empirically demonstrated. Because no validated measure currently captures adaptive pleasure across all four Four Kai domains, future studies should develop and validate appropriate measures of this construct. Such assessments might include domain-specific subjective experiences, such as post-sleep refreshment or restoration, satisfaction and comfortable satiety after eating, relief or comfort following bowel movements, and enjoyment, vitality, or refreshment during or after physical activity. These subjective measures could be evaluated alongside established objective and behavioral measures of sleep, dietary behavior, bowel function, and physical activity.

Longitudinal studies could clarify temporal relationships among these behaviors, whereas intervention studies could determine whether approaches targeting multiple Four Kai behaviors provide benefits beyond conventional single-behavior interventions. Analytical approaches such as network analysis and cross-lagged longitudinal models may be particularly useful for examining patterns of interrelationships and temporal associations among the Four Kai behaviors and their associations with adaptive pleasure, self-regulation, and daytime functioning. Such studies should also test whether adaptive pleasure and self-regulation mediate any observed effects on daytime functioning. Rather than seeking simply to confirm the proposed framework, future research should determine which relationships are supported, which require modification, and whether the Four Kai framework provides explanatory or practical value beyond existing approaches to sleep health and integrated lifestyle behaviors.

Ultimately, such evidence may clarify whether the Four Kai framework offers a useful conceptual and practical approach to promoting healthy daytime functioning, sustainable health behaviors, and well-being throughout childhood and adolescence.

## Figures and Tables

**Table 1:** Thematic Categories Used in the Descriptive Literature Mapping.

Thematic Categories	Scope
1. Sleep health	Studies on normal sleep characteristics, sleep duration, sleep quality, circadian rhythm, sleep hygiene, and healthy sleep promotion.
2. Sleep disorders	Studies focusing on sleep disorders, including obstructive sleep apnea, insomnia, narcolepsy, restless legs syndrome, parasomnias, circadian rhythm sleep disorders, and sleep problems associated with specific diseases.
3. Mental health	Studies examining relationships between sleep and psychological or psychiatric outcomes, including depression, anxiety, stress, emotional well-being, and behavioral problems.
4. Obesity/Metabolism	Studies investigating obesity, body composition, metabolic health, diabetes, insulin resistance, energy metabolism, or related endocrine outcomes in relation to sleep.
5. Physical activity/Exercise	Studies evaluating physical activity, exercise interventions, sports performance, sedentary behavior, or motor function in relation to sleep.
6. Screen time/Media	Studies examining screen exposure, smartphones, social media, video games, television, or other digital media in relation to sleep.
7. Diet/Nutrition	Studies focusing on dietary habits, nutrient intake, eating behaviors, meal timing, infant feeding, or nutritional interventions in relation to sleep.
8. Gut–brain axis	Studies investigating gastrointestinal symptoms including constipation, gut microbiota, digestive function, or gut–brain interactions in relation to sleep.
9. Cardiovascular health	Studies focusing primarily on blood pressure, cardiovascular disease, vascular function, or other cardiovascular outcomes associated with sleep.
10. School/ Daytime functioning	Studies examining academic performance, cognitive function, attention, daytime sleepiness, school attendance, learning, or other daytime functioning associated with sleep.
11. Family/Parenting/ Environment	Studies investigating parental factors, family environment, caregiving, socioeconomic conditions, home environment, or other environmental influences on sleep.
12. Integrated lifestyle	Studies simultaneously evaluating sleep together with multiple lifestyle behaviors or broader behavioral and environmental factors, including physical activity, sedentary behavior, diet, screen use, family-related interventions, and studies based on 24-h movement guidelines.
13. Other	Studies that did not fit into any of the above categories, including methodological studies, measurement tools, healthcare delivery, epidemiological methods, and basic physiological research.

**Table 2:** Distribution of Thematic Categories in Pediatric and Adolescent Sleep Studies, January–June 2022–2026.

Year/Category	2022	2023	2024	2025	2026	Total
Sleep health	21	32	32	28	26	139
Sleep disorders	10	22	24	34	48	138
Mental health	14	8	10	7	16	55
Obesity/Metabolism	6	10	10	15	13	54
Physical activity/Exercise	28	24	28	35	29	144
Screen time/Media	5	9	9	19	26	68
Diet/Nutrition	20	23	21	23	17	104
Gut–brain axis	7	6	13	5	14	45
Cardiovascular health	2	0	2	4	0	8
School/Daytime functioning	7	7	11	7	4	36
Family/Parenting/Environment	17	23	21	27	22	110
Integrated lifestyle	46	53	58	67	44	268
Other	2	0	8	5	12	27
Total	185	217	247	276	271	1196

Note: The thematic categories differ substantially in conceptual breadth. Accordingly, the frequencies shown in this table are intended as descriptive indicators of research themes and should not be interpreted as quantitative estimates of the relative importance, strength of evidence, or overall research activity of individual fields. In particular, the relatively large number of studies classified as Integrated lifestyle may partly reflect the broader scope of this category.

## Data Availability

No new data was created or analyzed in this study. Data sharing is not applicable to this article.
